# Implementation of distributed arithmetic-based symmetrical 2-D block finite impulse response filter architectures

**DOI:** 10.12688/f1000research.126067.1

**Published:** 2023-09-21

**Authors:** Pratyusha Chowdari Ch, J.B. Seventline

**Affiliations:** 1 Department of Electronics and Communications Engineering, Gokaraju Rangaraju Institute of Engineering and Technology, Hyderabad, 500090, India; 2 Department of Electrical, Electronics and Communication Engineering, GITAM University, Visakhaptnam, Andhra Pradesh, 530045, India

**Keywords:** Systolic architecture, block processing, distributed arithmetic, 2-D finite impulse response, symmetries in FIR filter

## Abstract

**Background:** This paper presents an efficient two-dimensional (2-D) finite impulse response (FIR) filter using block processing for two different symmetries. Architectures for a general filter (without symmetry) and two symmetrical filters (diagonal and quadrantal symmetry) are implemented. The proposed architectures need fewer multipliers because of the symmetry of the filter coefficients.

**Methods:** A distributed arithmetic (DA)- based multiplication method is used in the proposed architecture. A dual-port memory-based lookup table (DP-MLUT) is used in the multiplication instead of lookup-table (LUT) to reduce the area and power of the FIR filter. The filter's throughput is increased by using block processing. Memory reuse and memory sharing methods are introduced, which reduces the need for many registers and hence the circuit complexity. The architectures are written in Verilog Hardware Description Language and synthesized using Genus Synthesis tool-19.1 in 45nm technology with a generic library of Cadence vendor constraints. The synthesis tool generates the area, delay, and power reports. Power consumption of architectures is calculated with an image size of 64 X 64 and at 20 MHz frequency.

**Results:** Compared to existing architectures, the synthesis results show improvements in power, area, area delay product (ADP), and power delay product (PDP). The proposed MLUT-based 2-D block Quadrantal Symmetry Filter (QSF) for length 8 with block size 4 consumes 58.94% less power, occupies 59.5% less area, 48.44% less ADP and 47.78% less PDP compared to best existing methods.

**Conclusions:** A novel DA-based 2-D block FIR filter architecture with various symmetries is realized. Symmetry is incorporated into the filter coefficients to minimize the number of multipliers. The LUT size is optimized by odd multiples or even multiples storage techniques. Also, the overall area of the architecture is decreased by DP-LUT-based multipliers. The proposed filter architecture is area-power-efficient. It is best suited for applications that have fixed coefficients.

## Introduction

Many image and video processing applications, including image enhancement, template matching, image restoration, and video communication, use 2-D digital filters. ^1^^,^^2^ Finite impulse response (FIR) filters are preferred over infinite impulse response (IIR) filter when the numerical stability, ease of design and linear phase are the primary concerns. ^1^ Because 2-D FIR filters need numerous computations, the efficient structure design is challenging for researchers. In, ^1^ Parhi proposed a systolic structure for a 2-D FIR filter and suggested many techniques to optimize the implementation of 1-D and 2-D FIR and IIR filter architectures with more computational blocks. The block-based 2-D FIR filter banks consisting of separable and non-separable architectures with a significant reduction in memory are described. ^3^^,^^4^ In, ^3^ conventional multipliers, which consume power, are used for the convolution of input samples and filter coefficients, and there is no consideration of the internal architectures of symmetry filters.

The power-efficient and memory-efficient 2-D FIR filter architectures (FIRAs) are constructed with high-speed multipliers and parallel prefix modified carry look ahead adder (MCLAA). ^5^ The low area-memory-based non-symmetry type 2-D FIRA is proposed with a new multiplication technique. ^6^ In the above works, no symmetry concept is considered. The arithmetic computations are decreased by coefficient symmetry in the systolic filter architecture. ^7^^,^^8^ The low-power multimode architectures for 2-D IIR filters are designed and implemented with four symmetries. The critical path analysis is addressed for symmetry filters, but the architectures are implemented only for single input processing. Another single input processing-based quadrantal symmetry is implemented using the 2-D L _1_- technique to minimize the filter coefficients and hardware blocks. ^9^ Recently, Chowdari *et al.* ^24^^–^^26^ have proposed efficient implementation of DA based adaptive filter.

Mohanty *et al*. ^10^ proposed a 1-D block filter for narrowband applications using a Distributed Arithmetic (DA)-based reconfigurable filter for the software define radio SDR channelizer. Introduced the memory sharing concept to implement a 1-D finite impulse response (FIR) filter with a low area-power-delay. Several authors have implemented only DA-based 1-D filters. In recent years, DA techniques have attained great importance in FIR filter implementation to reduce the complexity of the architecture with high throughput and regularity. Kumar *et al*. ^11^^,^^12^ recently proposed block-based 2-D FIR and IIR filter architectures using DA with a memory-sharing approach but did not discuss the symmetry of coefficients. DA-based FIRAs are described in, ^13^^,^^14^ and the review of DA methods for cost-effective and efficient FIRAs is summarized. Park *et al*. ^15^ have suggested reconfigurable FIR architecture using DA.

In all the DA-based filter implementation schemes, the authors focused only on the decreasing adders' quantity and multiplier complexity. Memory complexity is one of the key factors while designing the filter, affecting power consumption and area. Many researchers have addressed the 1-D and 2-D filters using symmetry or block processing in filters. ^16^^,^^17^ Few researchers have realized the filter structures with Lookup Table (LUT)-based or DA multipliers without block processing or symmetry.

A new approach to memory-based DA multiplication is proposed by Meher *et al*. ^18^^,^^19^ This memory-based LUT (MLUT) multiplication approach is used to realize the 1-D FIR filters. The comparison analysis is presented with conventional multiplier-based filter architectures. Vinitha *et al.* ^6^^,^^20^ also developed the LUT-based multiplication and incorporated it into the filer architectures with fewer hardware blocks. Chiper *et al*. ^21^ suggested the dual-port concept in the MLUT-based DA multiplication rather than Single-Port LUT (SPLUT) multipliers. The modified memory-based multipliers are realized to implement an efficient filter architecture by Sharma *et al*. ^22^ Alawad *et al*. ^23^ presented a stochastic-based 2-D FIRA with low hardware complexity and high throughput. The probabilistic convolution theorem is used for the proposed non-separable systolic 2-D FIRA. The proposed work solves this problem within a predetermined accuracy range. The probability density function represents the 2-D input signal kernels by exploiting the convolution theorem. This well-known probabilistic convolution theorem replaces the expensive multipliers with simple adders. The memory storage complexity is also reduced by memory sharing and memory reuse. This work is more suitable for applications like perception-based image processing, which can inherently tolerate some computing inaccuracy.

The addressed points motivate developing and implementing the block-based 2-D FIRAs using various symmetries and multiplier-less DA-based approaches. In this research, two types of symmetries, diagonal symmetry and quadrantal symmetry, are considered to reduce the multipliers. The hardware in adders is increased by block processing in symmetry filters, although multipliers are more complex than adders. A novel MLUT multiplication approach is introduced in the 2-D block FIRAs. Two types of symmetries for 2-D FIRA and one non-symmetry filter are explored to decrease the number of multipliers. Conventional multipliers are replaced with MLUT multipliers to decrease each symmetry filter's power consumption, delay, and area.

The paper is organized as follows: The novel approach to designing the two types of symmetries and an optimized memory-based multiplication approach for 2-D FIRAs are discussed in background section. The next section describes the proposed 2-D FIRAs, and the individual symmetry filter architectures are explored according to the block processing using enhanced Dual-Port Memory-based LUT (DP-MLUT)-based multipliers.

## Background: Block-based design and symmetry of 2-D FIR filters

This section explains the various coefficient symmetry concepts and the MLUT multiplication approach to replacing normal multipliers.

### Block processing and memory reuse

In the digital filters, the block processing concept increases the throughput of the architecture. If the input block size is ‘ N ’, the filter produces ‘ N ’ outputs per one iteration, which means N -times throughput increases. The input matrix $X\left(n_{1},n_{2}\right)$ is needed at different systolic stages to generate a 2-D filter output $Y\left(n_{1},n_{2}\right)\;$ is of the length of the filter ( *L*). ^3^

$$X\left(n_{1},n_{2}\right)=\left[\begin{array}{ccc} \begin{array}{c} x\left(n_{1},n_{2}\right)\;\;x\left(n_{1},n_{2}-1\right)\\ \begin{array}{cc} x\left(n_{1}-1,n_{2}\right) & \;x\left(n_{1}-1,n_{2}-1\right) \end{array}\\ \begin{array}{cc} . & . \end{array} \end{array} & \begin{array}{c} ..\\ ..\\ .. \end{array} & \begin{array}{c} x\left(n_{1},n_{2}-L+1\right)\\ x\left(n_{1}-1,n_{2}-L+1\right)\\ . \end{array}\\ \begin{array}{cc} . & . \end{array} & .. & .\\ \begin{array}{cc} x\left(n_{1}-L+1,n_{2}\right) & x\left(n_{1}-L+1,n_{2}-1\right) \end{array} & .. & x\left(n_{1}-L+1,n_{2}-L+1\right) \end{array}\right]$$ (Eq. 1)

Let us consider $x\left(n_{1},n_{2}-m\right)$ is the ${\left(m+1\right)}^{\mathrm{th}}$ input of the 2-D FIR filter, $w\left(p,q\right)$ represents filter coefficients. The ${\left(m+1\right)}^{\mathrm{th}}$ output of the filter is expressed as ^3^:

$$Y_{m}\left(n_{1},n_{2}\right)={\left\{{\left\{x\left(n_{1}-p,n_{2}-m-q\right).w\left(p,q\right)\right\}}_{p=0}^{L-1}\right\}}_{q=0}^{L-1}$$ (Eq. 2)

$w\left(p,q\right)$ is expressed as equation (3). ^3^

$$w\left(p,q\right)=\left[\begin{array}{ccc} \begin{array}{c} \begin{array}{cc} w\left(0,0\right) & w\left(0,1\right) \end{array}\\ \begin{array}{cc} w\left(1,0\right) & \;w\left(1,1\right) \end{array}\\ \begin{array}{cc} . & . \end{array} \end{array} & \begin{array}{c} ..\\ ..\\ .. \end{array} & \begin{array}{c} w\left(0,L-1\right)\\ w\left(1,L-1\right)\\ . \end{array}\\ \begin{array}{cc} . & . \end{array} & .. & .\\ \begin{array}{cc} w\left(L-1,0\right) & w\left(L-1,1\right) \end{array} & .. & w\left(L-1,L-1\right) \end{array}\right]$$ (Eq. 3)

Thus, the 2-D FIR filter block output at each systolic stage is expressed as ^11^:

$$Y={\left\{G\left(n_{1},n_{2}\right).w_{p}^{T}\right\}}_{p=0}^{L-1}\;$$ (Eq. 4)

The filter coefficient vector required at each stage is expressed as ^11^:

$$w_{p}={\left[w\left(p,0\right)\;w\left(p,1\right)\;w\left(p,2\right)\ldots w\left(p,L-1\right)\right]}_{1\mathrm{XL}}$$ (Eq. 5)

Each iteration of the 2-D block FIRA needs the parallel calculation of a block of input samples and produces a block of output. At each systolic stage, a set of L−1 delayed inputs is required to generate a block of input. The input pixels at ${\left(p+1\right)}^{\mathrm{th}}$ the stage is represented by $G\left(n_{1},n_{2}\right)$ , which is given in matrix form as ^11^:

$$G\left(n_{1},n_{2}\right)=\left[\begin{array}{ccc} \begin{array}{c} \begin{array}{cc} x\left(n_{1}-p,n_{2}\right) & \;x\left(n_{1}-p,n_{2}-1\right) \end{array}\\ \begin{array}{cc} x\left(n_{1}-p,n_{2}-1\right) & \;x\left(n_{1}-p,n_{2}-2\right) \end{array}\\ \begin{array}{cc} . & . \end{array} \end{array} & \begin{array}{c} ..\\ ..\\ .. \end{array} & \begin{array}{c} x\left(n_{1}-p,n_{2}-L+1\right)\\ x\left(n_{1}-p,n_{2}-L\right)\\ . \end{array}\\ \begin{array}{cc} . & . \end{array} & .. & .\\ \begin{array}{cc} x\left(n_{1}-p,n_{2}-N+1\right) & x\left(n_{1}-p,n_{2}-N\right) \end{array} & .. & x\left(n_{1}-p,n_{2}-N-L+2\right) \end{array}\right]$$ (Eq. 6)

To facilitate parallelism, we further decompose the input pixel matrix $G\left(n_{1},n_{2}\right)$ and coefficient vector by a factor of s. The input pixel matrix $G\left(n_{1},n_{2}\right)$ is decomposed into $\frac{L}{S}$ sub matrices represented as $X_{p}^{q}$ of dimension $G\left(N\;X\;S\right)$ , and also the coefficient vector $w_{p}^{q}$ of dimension $\left(1\;X\;S\right)$ ; $0\leq q\leq \left(\frac{L}{S}\right)-1.$ Equation (4) is modified as ^11^

$${\left[Y\right]}_{\mathrm{NX}\;1}={\left\{{\left\{X_{p}^{q}\;{\left(w_{p}^{q}\right)}^{T}\right\}}_{q=0}^{\left(\frac{L}{s}\right)-1}\right\}}_{p=0}^{L-1}$$ (Eq. 7)

Where ^11^

$$X_{p}^{q}=\left[\begin{array}{ccc} \begin{array}{c} \begin{array}{cc} x\left(u,v\right) & \;x\left(u,v-1\right) \end{array}\\ \begin{array}{cc} x\left(u,v-1\right) & x\left(u,v-2\right) \end{array}\\ \begin{array}{cc} . & . \end{array} \end{array} & \begin{array}{c} x\left(u,v-2\right)\\ x\left(u,v-3\right)\\ .. \end{array} & \begin{array}{c} x\left(u,v-3\right)\\ x\left(u,v-4\right)\\ . \end{array}\\ \begin{array}{cc} . & . \end{array} & .. & .\\ \begin{array}{cc} x\left(u,v-N+1\right)) & x\left(u,v-N\right) \end{array} & x\left(u,v-N-1\right) & x\left(u,v-N-2\right) \end{array}\right]$$ (Eq. 8)

$w_{p}^{q}=\left[w\left(p,\mathrm{sq}\right)\;w\left(p,\mathrm{sq}+1\right)\ldots w\left(p,\mathrm{sq}+q-1\right)\right]$ , $u=\left(n_{1}-p\right)$ and $v=(n_{2}-\mathrm{sq}$ ). Equation (7) is re-writtenas

$$\;{\left[Y\right]}_{\mathrm{NX}\;1}={\left\{y_{p}^{q}\right\}}_{q=0}^{\left(\frac{L}{S}\right)-1}$$ (Eq. 9)

Where

$$y_{p}^{q}={\left\{X_{p}^{q}\;{\left(w_{p}^{q}\right)}^{T}\right\}}_{p=0}^{L-1}$$ (Eq. 10)

### Symmetry concepts of 2-D FIR filter structure

The symmetry concept is considered for the reduction of complex multipliers. In this paper, two types of symmetry, Diagonal Symmetry Filter (DSF) and Quadrantal Symmetry Filter (QSF) ^7^^,^^8^ for 2-D FIRAs, are studied and explored. The following transfer functions are used to design the two types of symmetries in the 2-D FIRA. ^7^^,^^8^

A) DSF 2-D FIR Filter: The transfer functions of DSF in magnitude response as $\left|H\left(z_{1},z_{2}\right)\right|=\left|H\left(z_{2},z_{1}\right)\right|,$ where $z_{1}=e^{j\theta_{1}}$ and $z_{2}=e^{j\theta_{2}}$ , $\forall \left(\theta_{1,}\theta_{2}\right)$ . The filter coefficients are related as $h_{\mathrm{ij}}=h_{\mathrm{ji}}$ for all i,j. Equation (11) expresses the transfer function of diagonal symmetry. ^17^

$$\frac{\;Y}{X}=\sum_{i=0}^{L}h_{\mathrm{ii}}z_{1}^{-i}z_{2}^{-i}+\sum_{i=0}^{L-1}\sum_{j=i+1}^{L}h_{\mathrm{ij}}\left(z_{1}^{-i}z_{2}^{-j}+z_{1}^{-j}z_{2}^{-i}\right)$$ (Eq. 11)

B) QSF 2-D FIR Filter: The QSF’s magnitude response is $\left|H\left(z_{1},z_{2}\right)\right|=\left|H\left(z_{1}^{-1},z_{2}\right)\right|$ , where $z_{1}=e^{j\theta_{1}}$ and $z_{2}=e^{j\theta_{2}}$ , $\forall \left(\theta_{1,}\theta_{2}\right)$ . The filter coefficient symmetry is given by $h_{\mathrm{ij}}=h_{\left(L-i\right)j}$ for all i,j. Equation (12) expresses the transfer function of the filter. ^17^

$$Y/X=\sum_{j=0}^{L}h_{\mathrm{uj}}z_{1}^{-u}z_{2}^{-j}+\sum_{i=0}^{L-1}\sum_{j=0}^{L}h_{\mathrm{ij}}\left(z_{1}^{-i}z_{2}^{-j}+z_{1}^{-\left(L-i\right)}z_{2}^{-j}\right)$$ (Eq. 12)

**Figure 1.**
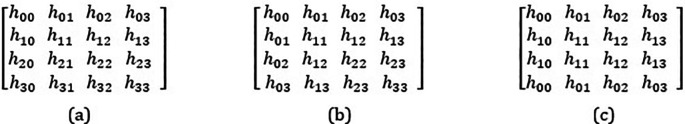
Filter coefficient matrices of (a) General filter (b) Diagonal Symmetry Filter (c) Quadrantal Symmetry Filter.

The general filter coefficients and two types of symmetry coefficient matrices are shown in Figure 1.

The proposed work implements two efficient symmetrical 2-D FIRAs and one generic filter architecture. Because of the symmetry of the filter coefficient, fewer multipliers are needed to design the filter.

### The LUT-DA multiplication process

A LUT is treated as memory in memory-based multiplication, and the precomputed outputs of filter coefficients are saved in the LUT. DA multiplication is the process of shifting and accumulating LUT output values. The input sample and coefficient are multiplied in the process of memory-based multiplication. The LUT memory can save 2 ^w^ possible values for the binary input of word length of w bits and a coefficient of bit length of c bits. In the process of standard LUT-based multiplication, it requires 2 ^w^ words to save the precomputed partial products in LUT.

Even multiples can be obtained from memory using left shift operations on odd multiples. This work uses (2 ^w^/2) words to save the odd multiples of coefficient C. This approach is shown for w = 4-bits of input sample in Table 1. In this table, the 8-address locations are stored by odd multiples of coefficient C, such as C, 3C, 5C, 7C, 9C, 11C, 13C, and 15C. Even multiples are evaluated using left shift operations of C, such as 2C, 4C, and 8C by 1-, 2-, and 3-times left shift operation to C, respectively. Next, 6C and 12C products are produced by a left shift of 3C; the remaining 10C is derived from 5C, and 14C is derived from 7C, respectively. The product output for the input sample consists of all zeros x=0000 produced by resetting the LUT.

**Table 1.** Memory-based Lookup Table – Distributed Arithmetic (MLUT-DA) multiplication approach. ^17^

Address A _2_ A _1_ A _0_	Name of the Word	Value to be stored in LUT	Input bits (w) x _3_ x _2_ x _1_ x _0_	Result	Number of shifts required	Control lines S _1_ S _0_
0 0 0	W0	C	0 0 0 1	C	0	0 0
			0 0 1 0	2 ^1^ × C	1	0 1
			0 1 0 0	2 ^2^ × C	2	1 0
			1 0 0 0	2 ^3^ × C	3	1 1
0 0 1	W1	3C	0 0 1 1	3C	0	0 0
			0 1 1 0	2 ^1^ × 3C	1	0 1
			1 1 0 0	2 ^2^ × 3C	2	1 0
0 1 0	W2	5C	0 1 0 1	5C	0	0 0
			1 0 1 0	2 ^1^ × 5C	1	0 1
0 1 1	W3	7C	0 1 1 1	7C	0	0 0
			1 1 1 0	2 ^1^ × 7C	1	0 1
1 0 0	W4	9C	1 0 0 1	9C	0	0 0
1 0 1	W5	11C	1 0 1 1	11C	0	0 0
1 1 0	W6	13C	1 1 0 1	13C	0	0 0
1 1 1	W7	15C	1 1 1 1	15C	0	0 0

**Figure 2.**
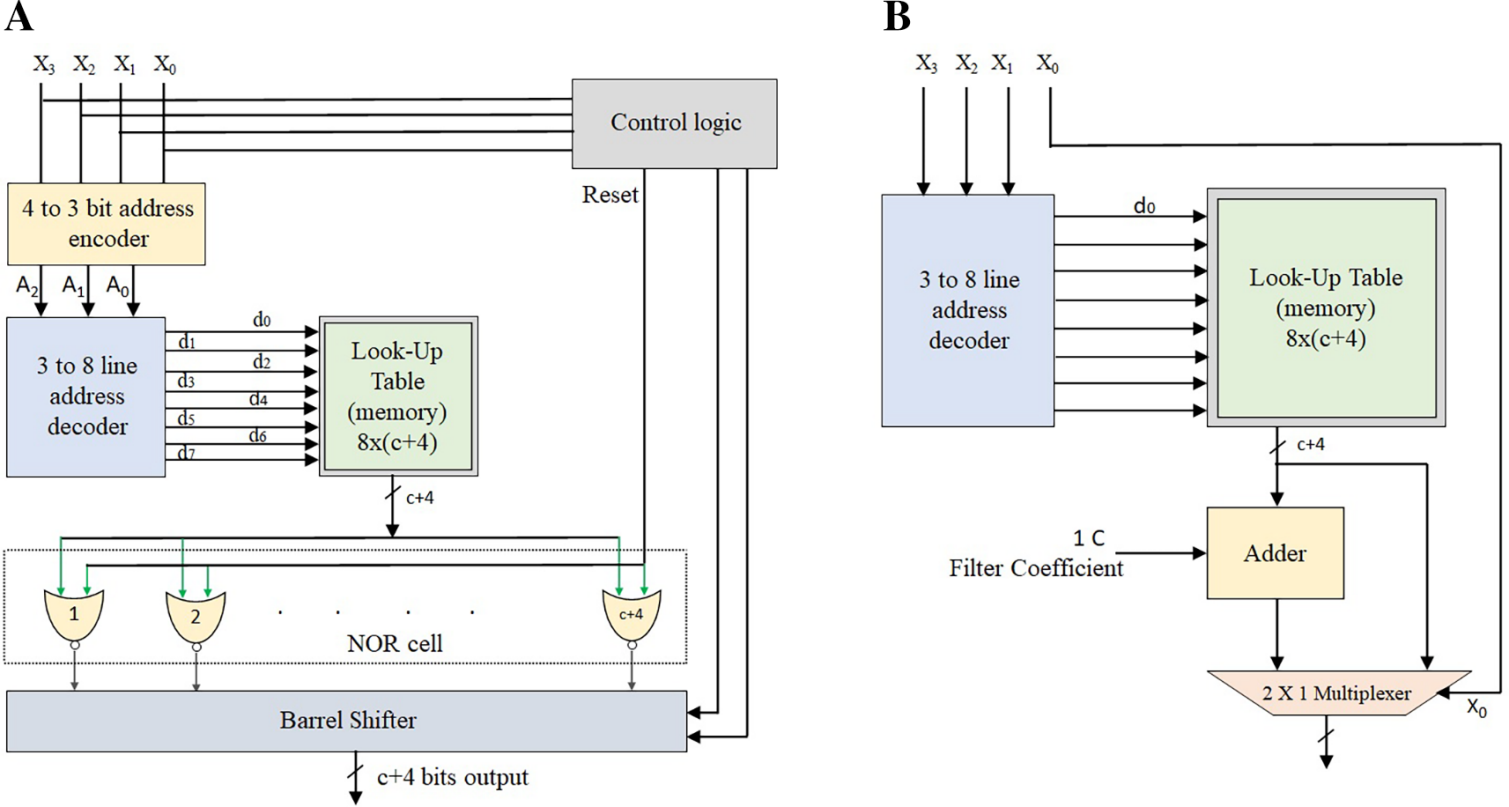
(A) Structure of conventional Memory-based Lookup Table (MLUT) multiplier for odd multiples (B) Modified MLUT multiplier for even multiples. Where MUX is multiplexer and RST is reset.

The single-port MLUT-DA multiplier is realized with reference to Table 1, as shown in Figure 2A. The structure has one 4-to-3 encoder block, one 3-to-8 decoder block, one control logic to produce Reset (RST), and control lines {S0, S1} to accommodate the shifts required for the computation of even multiples of coefficients such as 2C, 4C, 8C, 10C, 12C, and 14C. A maximum of three shifts are required, so two bits of control signals are contemplated in the structure.

Using a control logic block, the RST is formed from the applied input sample. It results in eight odd multiples of coefficients with $\left(c+4\right)$ bits. An extra 4 bits are essential to computing the highest odd multiple value 15C is precomputed and stored in the LUT. The decoder output corresponding location is read and fed to the NOR cell, which is made up of $\left(c+4\right)$ NOR gates with one common input of RST. The NOR cell outputs are shifted by a barrel shifter based upon the control signals {S0, S1} coming from the control logic. The barrel shifter has 2 × (c + 4) AOI (AND_OR_INVERT) gates or 2 × 1 multiplexers (MUXs). Finally, the barrel shifter output is the multiplication result of the input sample and coefficient.

The combinational logic expression of the 4-to-3 encoder, employed in the LUT multiplier, is indicated in equations (13), (14), and (15).

$$A_{0}=\bar{\bar{\bar{\left(x_{0}x_{1}\right)}.\bar{\left(x_{1}x_{2}\right)}}.(x_{0}+\bar{\left(x_{2}x_{3})\right)}}$$ (Eq. 13)

$$A_{1}=\bar{\bar{\bar{\left(x_{0}x_{2}\right)}.}(x_{0}+\bar{\left(x_{1}x_{3})\right)}}$$ (Eq. 14)

$$A_{2}=x_{0}.x_{3}$$ (Eq. 15)

where A _2_ A _1_ A _0_ are address bits derived from the actual input bits x _3_ x _2_ x _1_ x _0_. The control logic signals (RST and S0, S1) are given by equations (16), (17), and (18).

$$S_{0}=\bar{\left(x_{0}+\bar{\left(x_{1}+\bar{x_{2}}\right)}\right)}$$ (Eq. 16)

$$S_{1}=\bar{\left(x_{0}+x_{1}\right)}$$ (Eq. 17)

$$\mathrm{RST}=\bar{(x_{0}+x_{1}}).\bar{\left(x_{2}+x_{3}\right)}$$ (Eq. 18)

In very large scale integration (VLSI) design, the conventional multipliers consume more power and occupy more area, whereas the LUT-based multipliers save area and power consumption. Hence, a further reduction in the hardware is achieved by LUT-DA multipliers.

In the LUT, only the even multiples of the coefficients are saved. Hence, only 2 ^w^/2 words are required instead of all 2 ^w^ words. Even multiples can be translated into odd multiples by adding one filter coefficient magnitude. The barrel shifter and encoder blocks are not required for this modified multiplier, and one 2 × 1 MUX is required to choose the odd or even-multiple coefficients. Table 2 depicts the even multiples storing technique for w = 4. The even values of constant-coefficient $\left\{0,2C,4C,\ldots ,12C,14C\right\}$ are precomputed corresponding to $\left\{x_{3}x_{2}\;x_{1}\right\}$ using the 3-to-8 decoder and saved in the 8-LUT locations. The other input for the 2 × 1 MUX is the LUT even output, and the other input is the odd output from the adder. The selection lines of the MUX are the least significant bit LSB-bits of the input sample $x_{0}$ . Whether the coefficients are even or odd multiples depends on the input sample’s LSB bit. Figure 2B represents the modified LUT-based multiplier.

**Table 2.** The Memory-based Lookup Table (MLUT) multiplier using even multiples.

Name of the Word	Address x _3_ x _2_ x _1_	Value to be stored in LUT	input bits (w) x _3_ x _2_ x _1_ x _0_	Result
W0	0 0 0	0	0 0 0 0	0
			0 0 0 1	1C
W1	0 0 1	2C	0 0 1 0	2C
			0 0 1 1	3C
W2	0 1 0	4C	0 1 0 0	4C
			0 1 0 1	5C
W3	0 1 1	6C	0 1 1 0	6C
			0 1 1 1	7C
W4	1 0 0	8C	1 0 0 0	8C
			1 0 0 1	9C
W5	1 0 1	10C	1 0 1 0	10C
			1 0 1 1	11C
W6	1 1 0	12C	1 1 0 0	12C
			1 1 0 1	13C
W7	1 1 1	14C	1 1 1 0	14C
			1 1 1 1	15C

Likewise, odd multiples of the coefficient can be saved in an improved LUT-DA multiplier by using a subtractor to generate the required even multiples. In the proposed work, the SPLUT multiplier is converted into a DPLUT multiplier using the DA approach. When the input sample bits are more, the dual-port memory helps decrease the LUT size. The common filter coefficient is multiplied simultaneously with two separate input samples using a DPMLUT-based multiplier. The following section explains how the proposed filters use an improved MLUT-DA multiplier with even multiples storage.

## Methods

### Proposed architectures of block-based 2-D FIR filters

**Figure 3.**
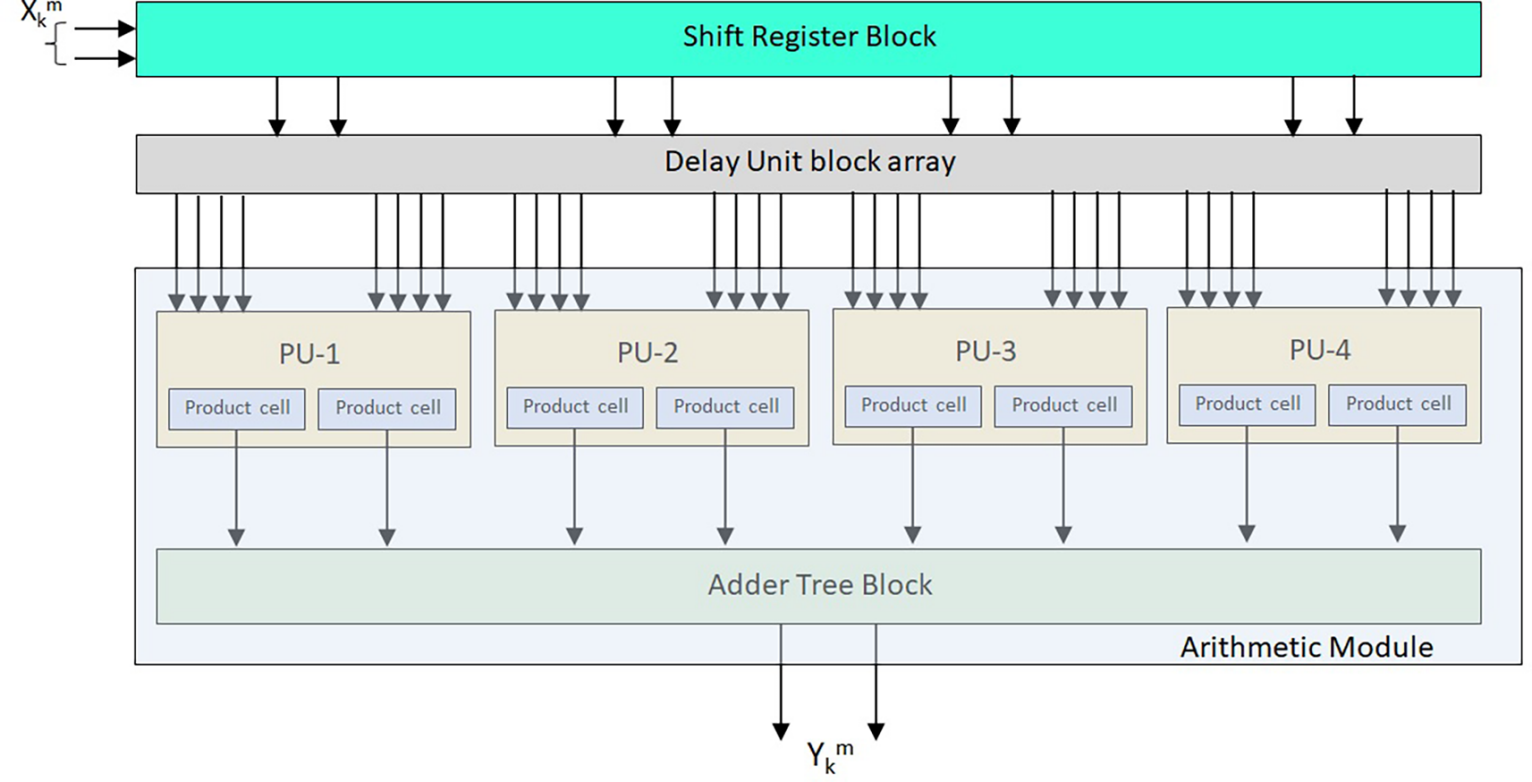
Conventional 2-D finite impulse response filter architecture (FIRA) for L = 4 with N = 2. Where PU is Processing Unit, $X_{k^{m}}$ is input and $Y_{k^{m}}$ is output.

The block-based 2-D FIRA is shown in Figure 3 for L = 4, with N = 2 without any symmetry, and is considered a general filter. The input samples {x _k_^0^, x _k_^1^} are from the same row of the image input matrix given to the shift register unit (SRU) array, and input samples are given in serial order, block by block and row by row. The SRU array contains $\left(L-1\right)$ SRUs, each with L shift registers with M words. Here, SRU1 is termed as {SR1, SR2}. Likewise, SRU2 and SRU3 are placed in an array form considered the SRU array for order L = 4. Each L - Delay Unit Block (DUB) produces NL samples by applying each set of past N and present samples to the total L sets.

**Figure 4.**
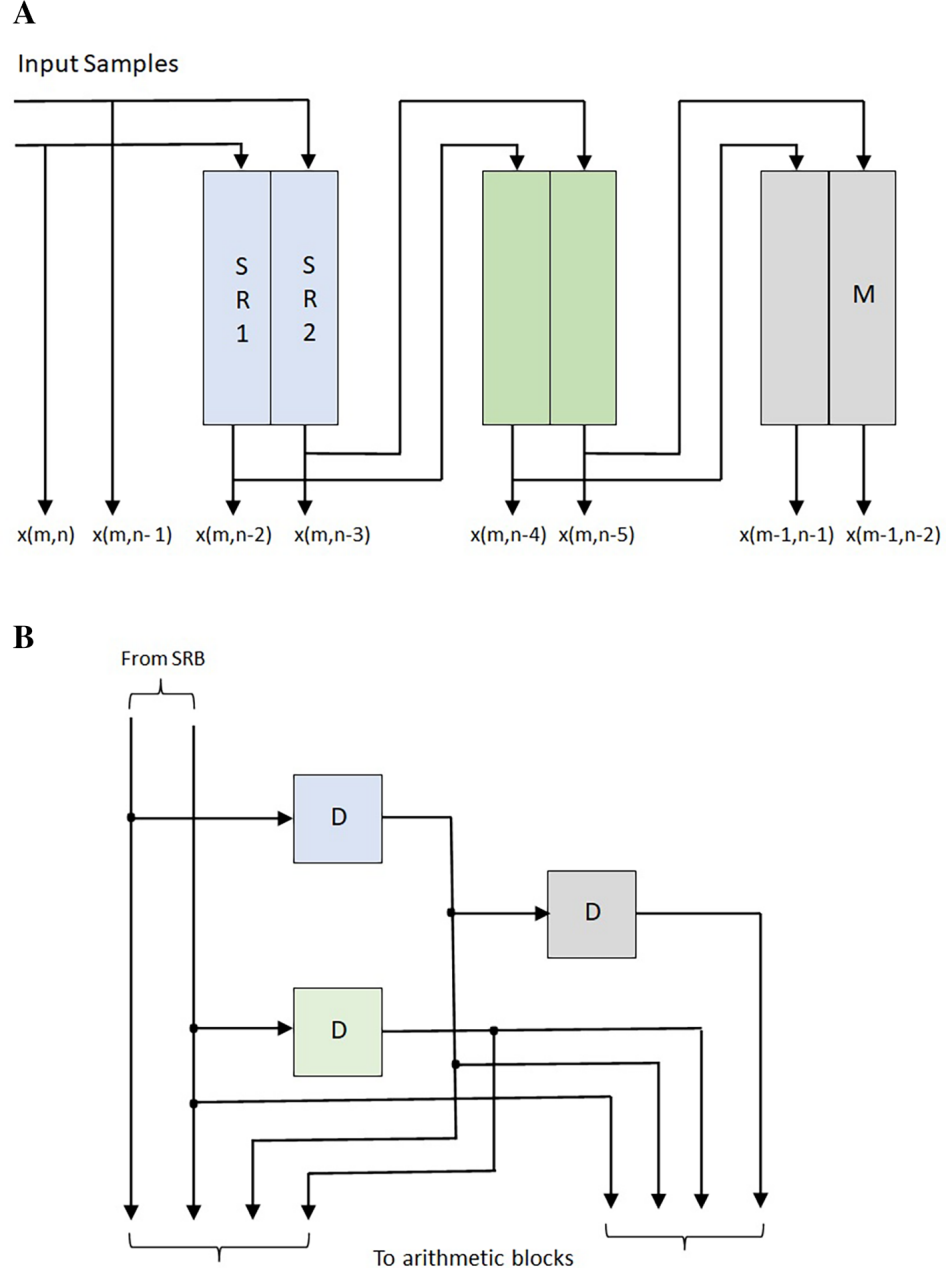
(A) Shift register unit (SRU) Array 2 (B) Delay Unit Block for L = 4 with N = 2.

The input block of *L* input samples from the image matrix $\left(M\times M\right)$ are applied as present inputs. The (L−1 ) SRU array receives these parallel inputs. Figure 4A represents the structure of SRU using the L number of registers. The present input sample and the past input sample blocks are applied to the N *-*DUBs of the DUB array. Each DUB consists of (L−1 ) flipflops. It produces the present and past samples required for block processing. As shown in Figure 4B, each DUB generates LN samples. The *L*-DUBs give the L×LN of input samples to the filter’s arithmetic module.

*Structures of block-based symmetric 2-D FIR filter arithmetic modules*

This section explores two symmetry-type 2-D FIR filters and one general filter of L = 4 with N = 2.

*General filter structure with MLUT multipliers*

The arithmetic module of the general filter architecture is realized by the L number of Processing Units (PU) and an Adder Tree (AT) block, which receives LN samples from DUB.

Each PU block is constructed by N number of Product Cells (PC), which are used to multiply the input sample by the corresponding filter coefficients. Generally, the product is done by conventional multipliers. MLUT multipliers are used in place of these power-hungry conventional multipliers. At last, the AT adds the outputs of the PU block and generates the N filter outputs corresponding to the N block of inputs.

**Figure 5.**
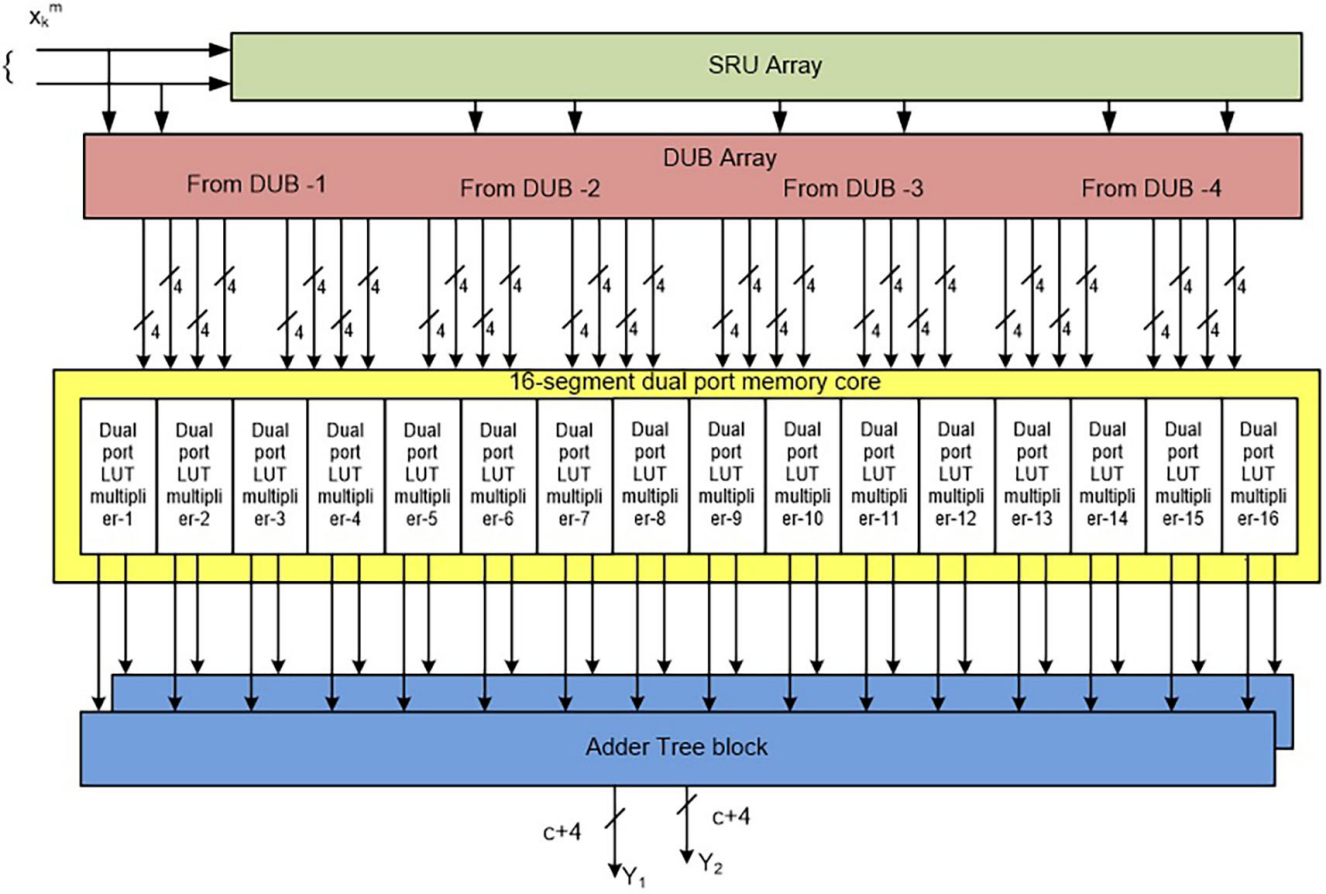
General 2-D filter architectures (FIRA) with dual-port look-up table (DPLUT) multipliers. SRU, shaft register unit; DUB, Delay Unit Block; LUT, look up table.

This general filter architecture is modified by DPMLUT multipliers, as presented in Figure 5. In this architecture, the inputs multiplied with the common filter coefficients are given to a DPLUT-multiplier. Hence, a total of $\left(L\times L\right)$ DPLUT multipliers are needed to process the complete multiplication of input samples of L = 4 and filter coefficients. $2\left(L\times L\right)$ multipliers are needed if SPLUT-based multipliers are used. The DPLUT-based multipliers save 50% of the area compared to SPLUT multipliers. Each DPLUT-based multiplier produces the L number of filter outputs. Total L memory multipliers generate $\left(L\times L\right)N$ number of outputs, and these are parallelly added by N -AT blocks and give N outputs with a size of (c+4 *)-*bits.

In this work, the multiplier quantity is decreased by symmetry in the filter coefficients. Two different symmetries are described in this section, and these symmetry filters can be used to design circular symmetry, fan-type and diamond filters.

*Structure of 2-D FIR Diagonal Symmetry Filter (DSF)*

**Figure 6.**
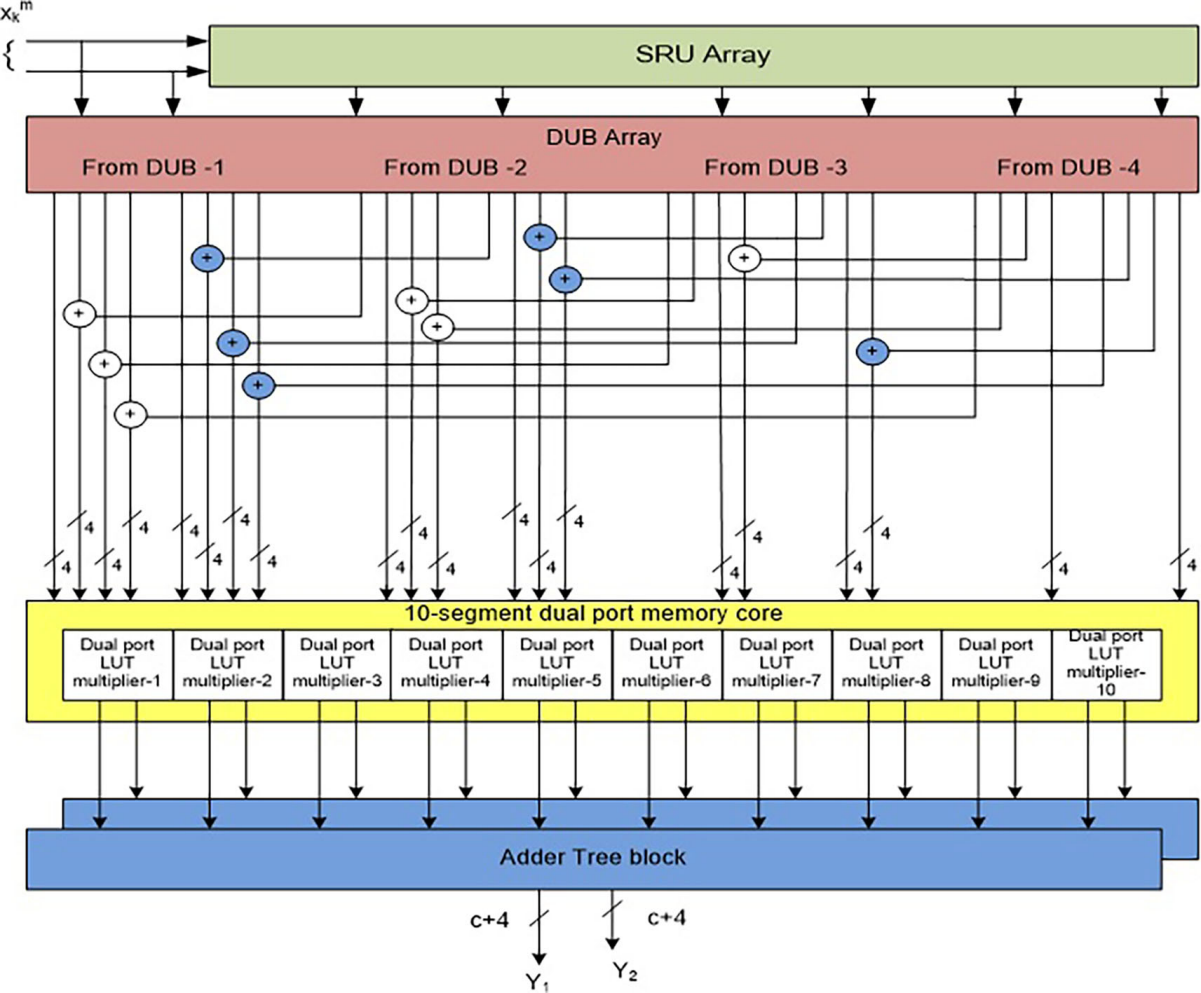
Structure of a diagonal symmetry 2-D Finite Impulse Response (FIR) filter with dual-port look-up table (DPLUT) multipliers. SRU, shaft register unit; DUB, Delay Unit Block; LUT, look up table.

In the DSF coefficient matrix, the sixteen coefficients are reduced to ten, such as { $h_{00},h_{01},h_{02},h_{03},h_{11},h_{12},h_{13},h_{22},h_{23},h_{33}\}$ for L = 4 as shown in Figure 1B. Figure 6 represents the arithmetic module of the DSF-based 2-D FIRA, and it is designed by diagonal symmetry. Before the multiplication process, the input samples to be multiplied with common filter coefficients are added.

For the one input of N = 2, seven adders are required to accumulate symmetry input samples. The seven highlighted colored adders indicate the adders for the other input sample. The adder is a simple block than the multiplier. The diagonal symmetry filter requires $\left(2L+2\right)N\;$ multipliers instead of $\left(L\times L\right)N,$ but extra $\left(L-1\right)N\;$ adders are required. Next, these $\;\left(2L+2\right)N\;$ multipliers are only designed for $\left(2L+2\right)N/2$ DPLUT-based multipliers. Hence, half of the area is optimized. Finally, all the multiplier output samples are accumulated by N - AT blocks to produce N outputs.

Because multipliers are responsible for most of the power consumption, DPLUT-based multipliers are used to optimize them. Hence, ten DPMLUT multipliers are needed to produce the N = 2 outputs from the diagonal symmetry filter. The DSF architecture for L = 4 with N = 2 needed 20 individual SPLUT multipliers.

**Figure 7.**
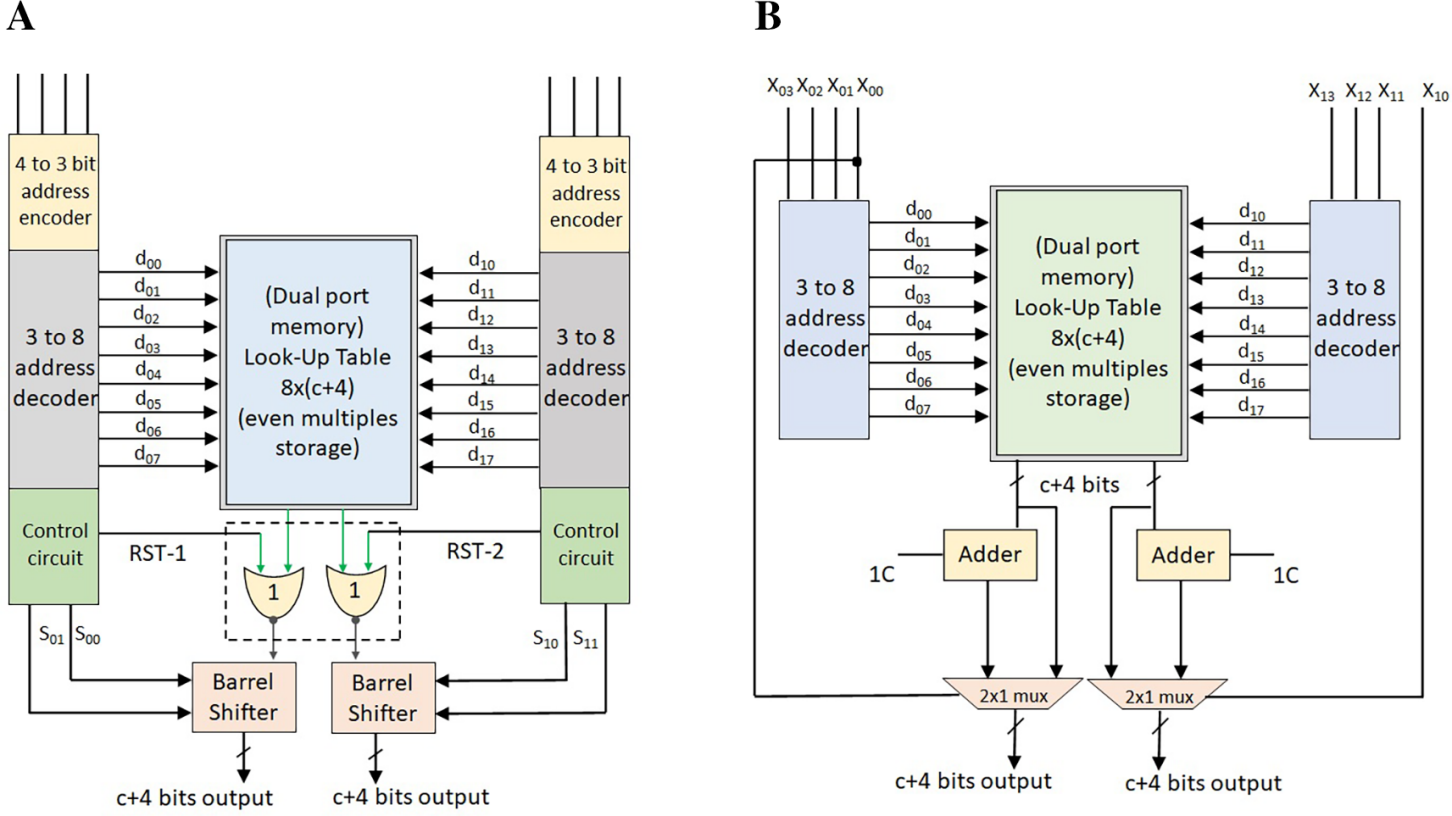
(A) Conventional dual-port look-up table (DPLUT) multiplier (B) Modified DPLUT multiplier. RST, reset.

DPLUT decreases the LUT size for input samples with greater bit lengths by adding an additional shifter. Because of parallel block processing, two inputs are multiplied with the common filter coefficient in a 2-D FIR filter. This concept can be used to replace two SPLUT multipliers with a single DPLUT multiplier. The internal structure of the conventional DPLUT-based multiplier and the modified DPLUT-based multiplier are shown in Figure 7A and B.

The common filter odd coefficient multiples are precomputed and placed in the LUT memory. According to the input bits, the address of the location in the LUT is determined by the address encoder and address decoder. DPLUT fetches the corresponding locations based on the given addresses of two ports and provides two parallel outputs. Furthermore, each output is shifted by barrel shifters after passing through the corresponding NOR gate. The control lines for shifting are generated from the input sample bits handled by some control circuit logic, as explained earlier.

This conventional DPLUT-DA multiplier has been revised, shown in Figure 7B for w = 4 bits of input sample using even multiples storage in LUT. It can be observed that the modified even multiples storage LUT-DA multipliers need less memory and area. Control logic for RST, barrel shifter, NOR cell, 4-to-3 encoder, and control signals of barrel shifter $\left\{s_{o},s_{1}\right\}$ are not needed to enhance the DPLUT multiplier, and this feature reduces area further.

**Figure 8.**
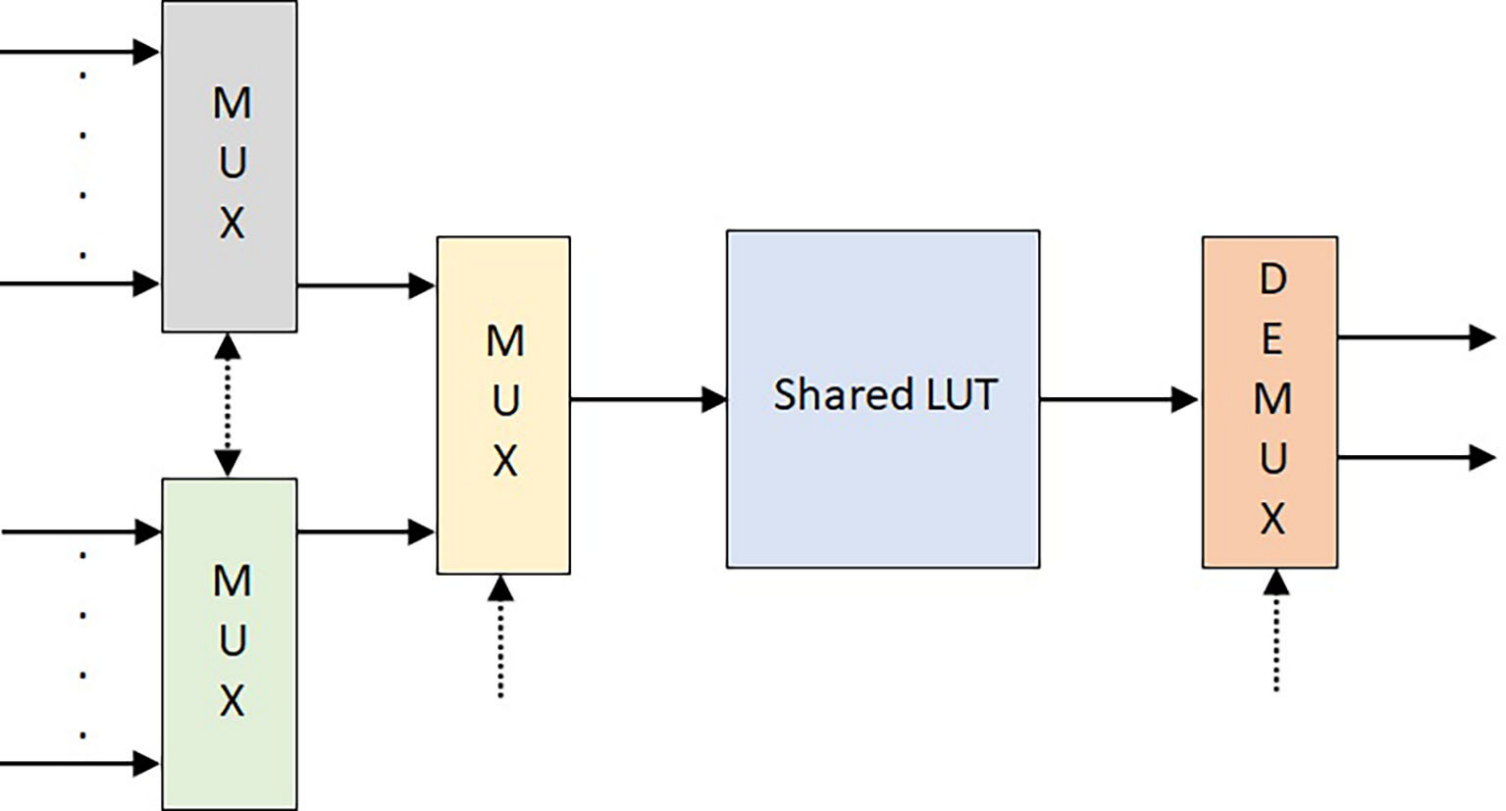
Dual-port look-up table (DPLUT) control logic. MUX. Multiplexer; LUT, look up table.

The conversion of SPLUT into DPLUT is a critical task. The common filter coefficients stored in the LUT must be shared by two inputs simultaneously. For this, the control logic is introduced related to the clock signal to choose the address locations with a slight delay. Figure 8 represents the control logic using multiplexers for a DPLUT-DA multiplier.

*Structure of a 2-D FIR Quadrantal Symmetry Filter (QSF)*

**Figure 9.**
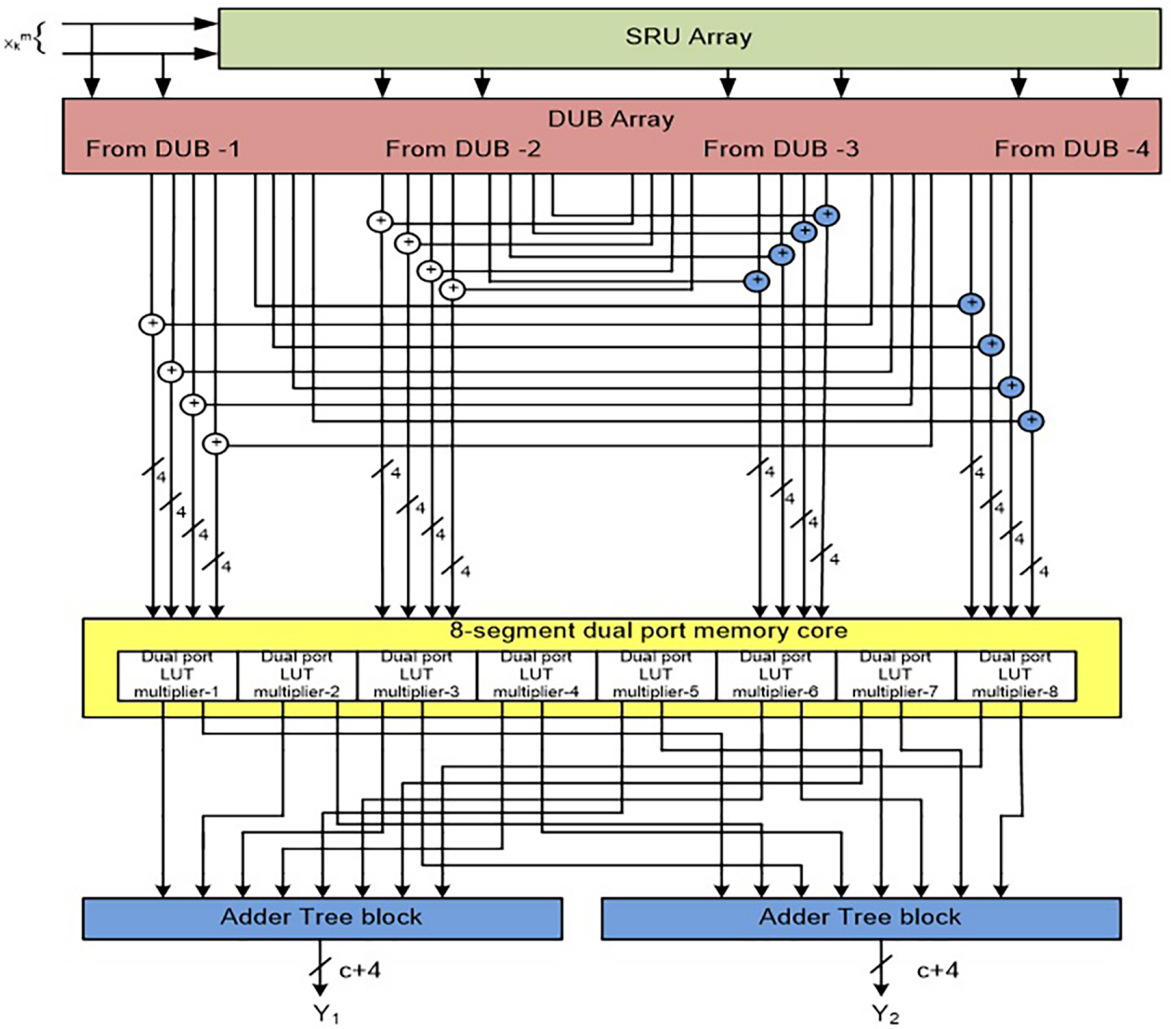
Structure of 2-D Finite Impulse Response (FIR) Quadrantal Symmetry Filter (QSF) with dual-port look-up table (DPLUT) multipliers. DUB, Delay Unit Block; SRU, shaft register unit; LUT, look up table.

The QSF consists of eight unique filter coefficients are given as { $.h_{00},h_{01},h_{02},h_{03},h_{10},h_{11},h_{12},h_{13.}$ }. Figure 9 represents the architecture of QSF for L = 4 with N = 2. A total of 16 SPLUT multipliers are needed for this structure, and it is modified with eight DPLUT multipliers to produce N -block outputs.

The summary of the number of single-port and dual-port multipliers needed for each symmetry is presented in Table 3.

**Table 3.** The multipliers count for constructing various symmetry filters for L = 4 with N =2.

Name of the filter	Single-port look-up table (SPLUT) multipliers	Dual-port look-up table (DPLUT) multipliers
General filter	32	16
Diagonal Symmetry Filter DSF	20	10
Quadrantal Symmetry Filter QSF	16	8

*Experiment/validation*

This section analyzes the implementation and results for the proposed 2-D FIRAs. Multipliers, registers, and adders construct the architecture of the proposed filters. The hardware block's complexity depends on the filter input sample bits, length L , input block size N , and filter coefficients. Hence, DSF and QSF symmetry-based 2-D FIR filters are designed and explored to reduce the quantity of the multipliers. Next, the multiplier architectures are optimized by dual-port even multiples storage LUT- based multipliers. The architectures are synthesized using the Genus Synthesis tool-19.1 in 45nm technology with a generic library of Cadence vendor constraints. There is a free synthesis tools available like Xilinx Integrated Synthesis Environment, which can be used instead of Genus Synthesis tool in Cadence to replicate our methods. Power consumption of architectures is calculated with an image size of 64 X 64 and at 20 MHz frequency. The synthesized results (reports in *Underlying data* ^27^) have been analyzed and compared with the existing architecture’s results. All Verilog code associated with the work is available in *Software availability.* ^28^

## Results and discussion

The data associated with the results is available in *Underlying data.* ^27^ Table 4 presents synthesis results of two individual types of symmetry 2-D FIR filters and general filters for L = 4 with N = 2.

**Table 4.** Power, delay, and area parameters of different multipliers for various symmetry filters for L = 4, N = 2 and w = 4. SPLUT, Single-port look-up table; DPLUT, Dual-port look-up table; DSF, Diagonal Symmetry Filter; QSF, Quadrantal Symmetry Filter.

Name of the Filter	Normal Multipliers			SPLUT Multipliers			DPLUT Multipliers		
	Power (mW)	Delay (ns)	Area (μm ^2^)	Power (mW)	Delay (ns)	Area (μm ^2^)	Power (mW)	Delay (ns)	Area (μm ^2^)
General Filter	1.2815	14.601	29711	0.9972	11.875	26122	0.775	10.22	23722
DSF	1.0624	14.209	22652	0.7212	11.432	19116	0.6981	10.238	17999
QSF	1.0103	14.112	21764	0.6996	11.228	17694	0.5591	10.623	14389

**Figure 10.**
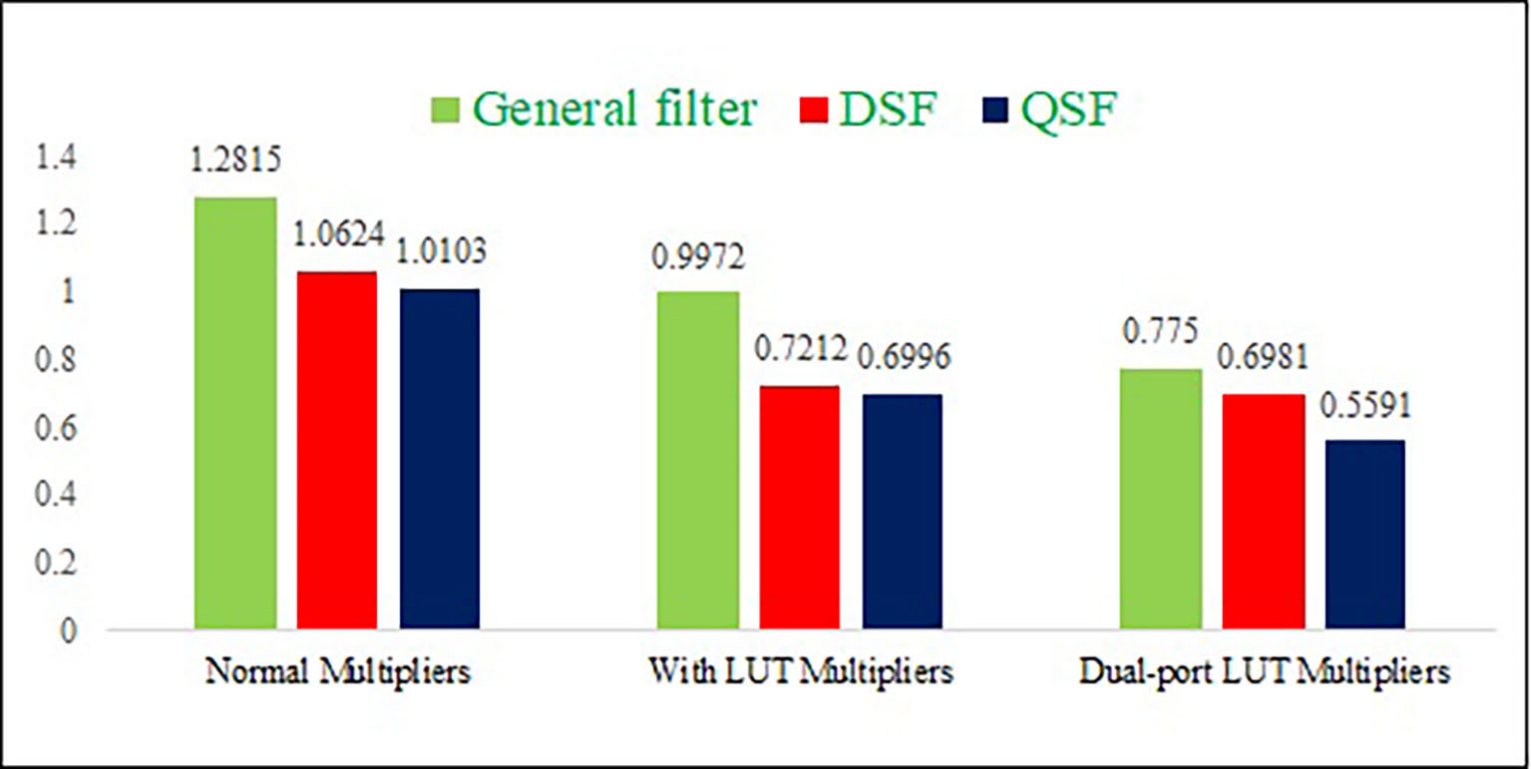
The power consumption comparison of different proposed 2-D Finite Impulse Response filters with different multiplier techniques. Where DSF is diagonal symmetry filter, QSF is quadrantal symmetry filter and LUT is look up table.

**Figure 11.**
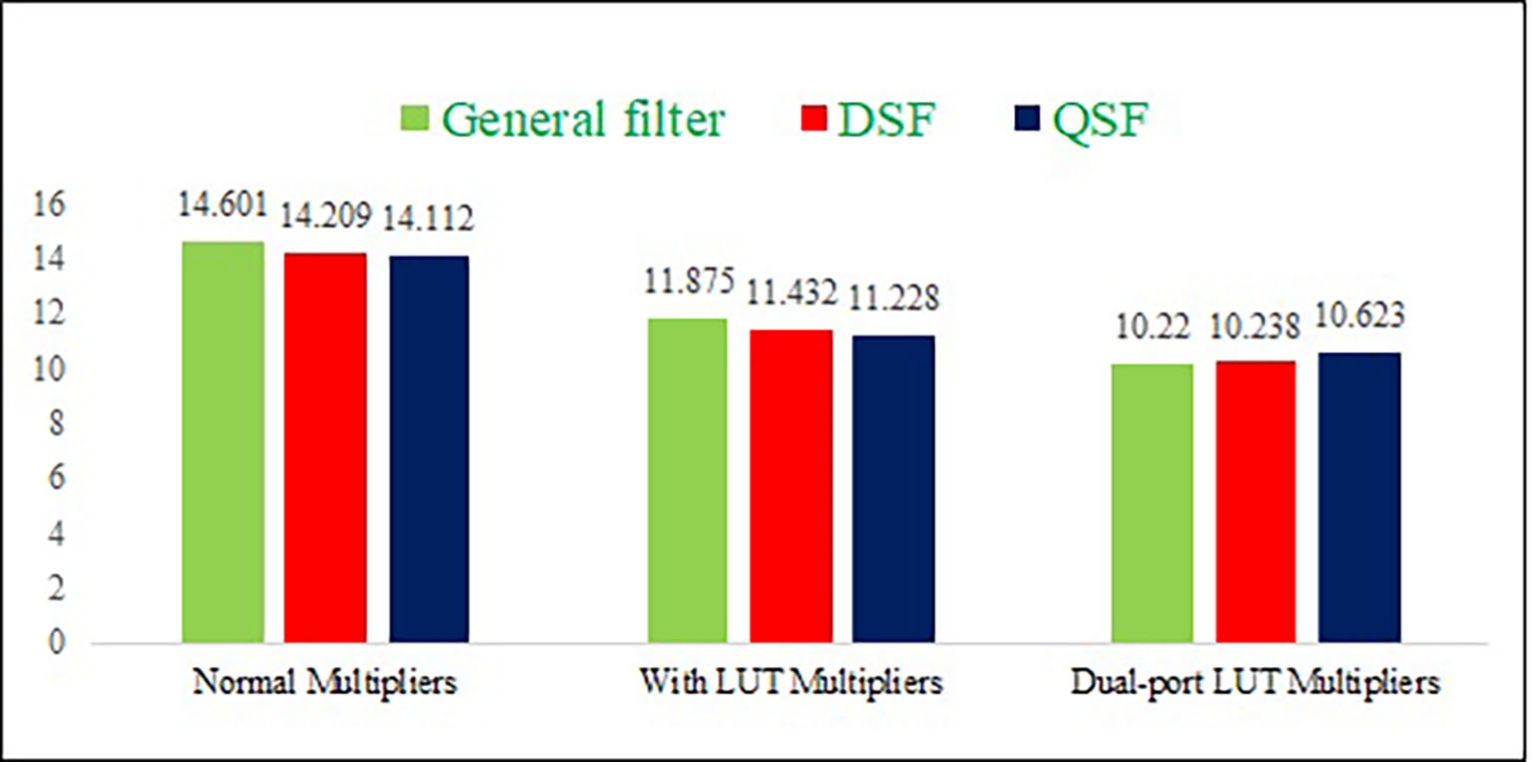
The delay comparison of different proposed 2-D Finite Impulse Response filters with different multiplier techniques. Where DSF is diagonal symmetry filter, QSF is quadrantal symmetry filter and LUT is look up table.

**Figure 12.**
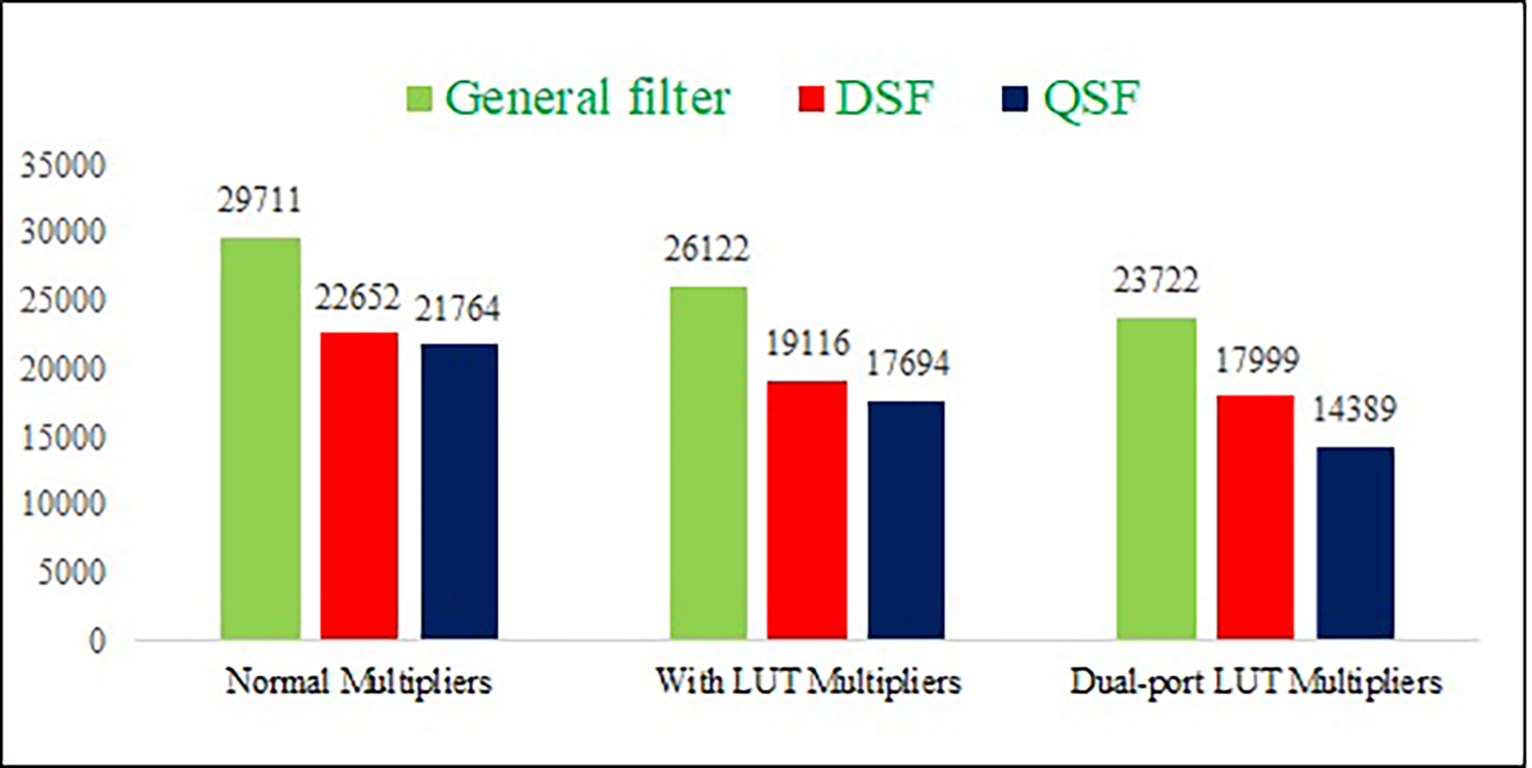
The area comparison of different proposed 2-D Finite Impulse Response filters with different multiplier techniques. Where DSF is diagonal symmetry filter, QSF is quadrantal symmetry filter and LUT is look up table.

The power consumption, delay, and area results are represented in graphs, as shown in Figures 10, 11, and 12, respectively. The proposed DPLUT-based 2-D FIR DSF-filter architecture needs 20.54% and 5.84% less area than normal and SPLUT multiplier-based filter architectures. 34.2% and 3.2% of power savings are obtained by the proposed filter architecture compared to the normal and single-port multiplier-based filter architectures, respectively. The proposed DSF architecture is 27.9%, and 10.4% has less delay than normal multiplier and SPLUT-based architectures. Similarly, the proposed QSF 2-D FIRA power is decreased by 44%, 20%, than normal and SPLUT multipliers, the area is decreased by 33%, 18.6%, and delay is decreased by 24.7%, 5.3% than normal and SPLUT multipliers, respectively.

The filter architectures of 2-D FIR with two symmetries and one general filter are implemented by block processing and dual-port memory-based multipliers. Here, the memory reuse concept is used to get the filter outputs, and memory saving is obtained. The VLSI performance metrics, such as area, delay, and power values of the proposed filters, are compared for input bits w = 4 and 8 in Table 5.

**Table 5.** Comparison of proposed architectures for L = 4, N = 2, w = 4 and 8.

Name of the Filter	Power (mW)		Delay (ns)		Area (μm ^2^)	
Input bits	w = 4	w = 8	w = 4	w = 8	w = 4	w = 8
General Filter with dual port lookup table multipliers	0.775	0.854	10.22	10.22	23722	28963
Diagonal symmetry filter with dual port lookup table multipliers	0.698	0.7458	10.238	10.22	17999	20186
Quadrantal symmetry filter with dual port lookup table multipliers	0.559	0.6253	10.623	10.8	14389	16453

**Figure 13.**
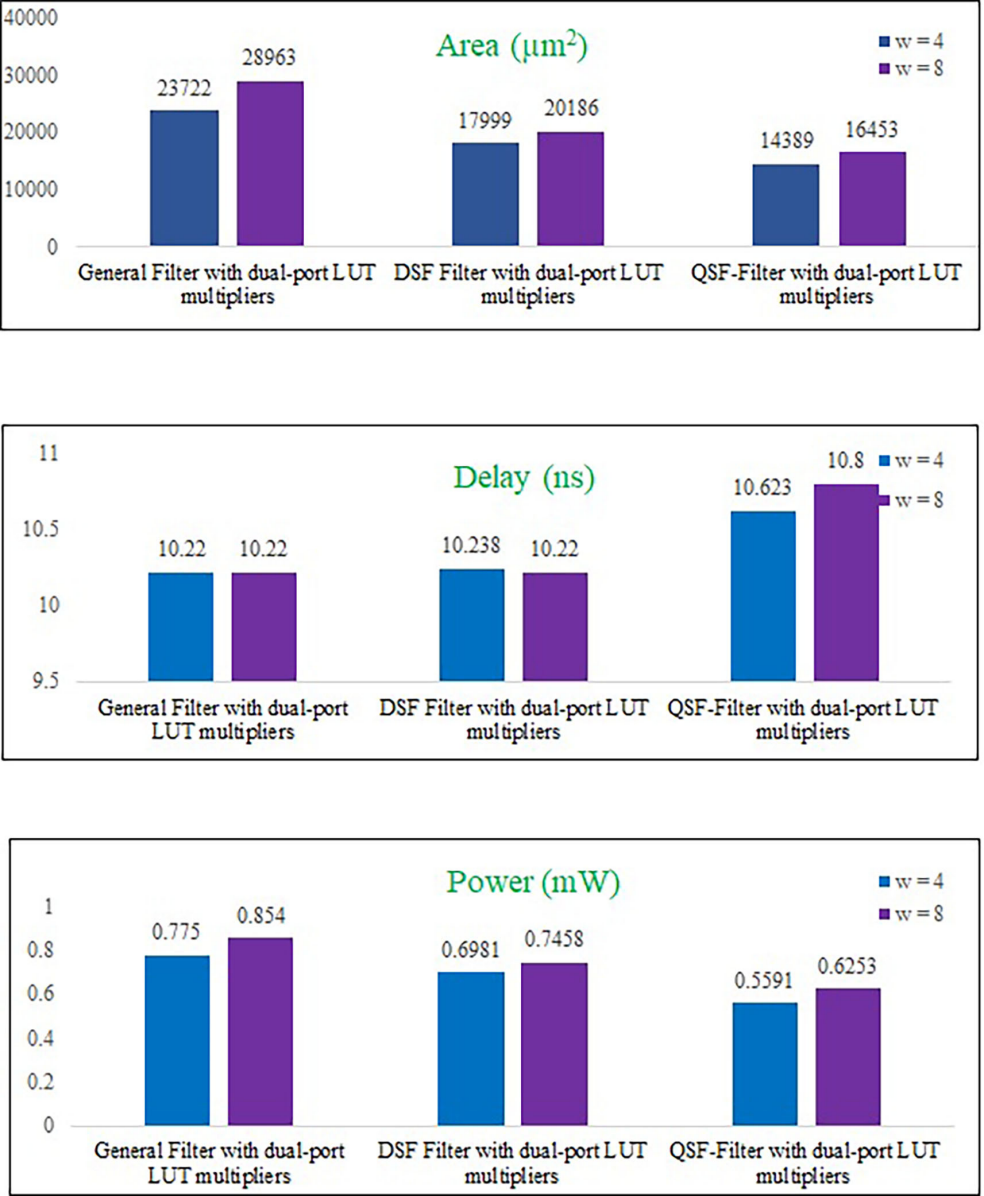
The area, delay, and power consumption comparison of proposed filters. DSF, diagonal symmetry filter; QSF, quadrantal symmetry filter; LUT, look up table.

The area of the proposed DSF and QSF symmetry filter architectures is reduced by 24.1% and 39.3% to the general 2-D FIRA for w = 4. The power-saving obtained by DSF and QSF is 9.9% and 27.8% less than the general filter architecture. The delay values of DSF, QSF and general filter are almost the same. Figure 13 represents the comparison of area, delay, and power consumption of the proposed DPLUT-based 2-D FIRAs for w = 4 and 8. It can observe that the VLSI performance metrics increase correspondingly when the filter's input sample bits increase.

for w = 4 and 8. Where DSF is diagonal symmetry filter, QSF is quadrantal symmetry filter and LUT is look up table. The proposed symmetry 2-D FIR filters with DPLUT-based multipliers are compared to previous works. The performance metrics obtained from the synthesis tool are tabulated in Table 6.

**Table 6.** Comparison of the proposed filters with existing filter for L = 8 with N = 4. DSF, Diagonal Symmetry Filter; QSF, Quadrantal Symmetry Filter; ADP, area delay product; PDP, power delay product.

Architecture	Area (μm ^2^)	Delay (ns)	Power (mW)	ADP (μm ^2^. ms)	PDP (mW. ns)
Alawad et al. [44]	489271	14.16	7.96	6.928	112.71
Mohanty et al. [45]	791361	16.25	5.0934	12.85	82.76
Kumar et al. [48]	405825	8.72	4.13	3.53	36.01
**Proposed Filter (DSF)**	**183457**	**11.258**	**1.8793**	**2.057**	**21.157**
**Proposed Filter (QSF)**	**164052**	**11.091**	**1.6955**	**1.82**	**18.804**

The proposed filter architecture implementation is extended for L =8 with N = 4. The 2-D FIRA for L =8 with N = 4 is also compared with the state-of-the-art works in Table 6. It can be observed that the proposed architecture is improved in terms of power, area, delay, ADP, and PDP than existing architectures.

**Figure 14.**
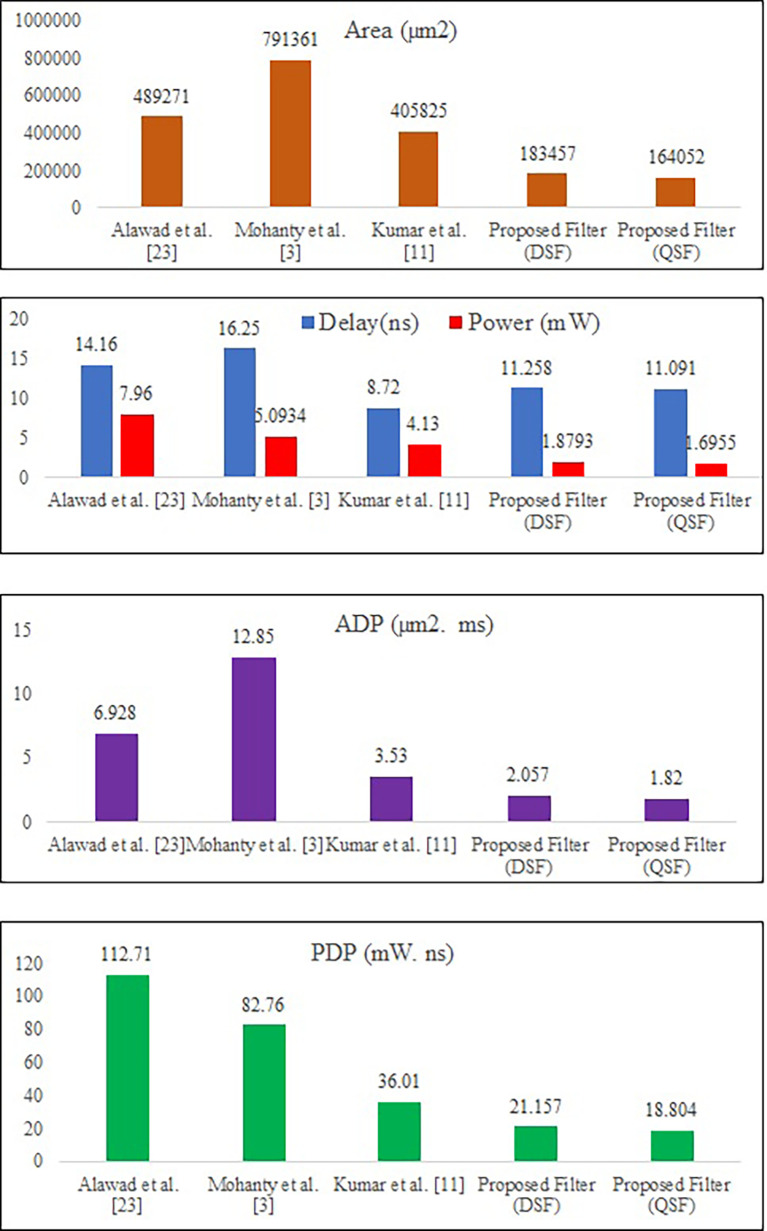
Area, delay-power consumption, ADP, and PDP comparison of proposed DSF and QSF architectures with existing architectures for L = 8 with N = 4.

A graphical comparison of results of rea, power consumption, delay, ADP, and PDP of the proposed structure with existing filter architecture for L = 8 with N = 4 is shown in Figure 14.

The proposed MLUT-based 2-D block DSF filter for L = 8 with N = 4 requires 62.50%, 76.81%, and 54.79% less area compared to [23], [3], and [11], respectively. It has 20.49%, and 30.72% less delay compared to [23], and [3], respectively. It consumes 76.39%, 63.10%, and 54.49% less power than [23], [3], and [11], respectively. It has 70.3%, 83.99%, and 41.72% less ADP than [23], [3], and [11], respectively. It has 81.22%, 74.43%, and 41.24% less PDP compared to [23], [3], and [11] respectively.

The proposed MLUT-based 2-D block QSF filter for L = 8 with N = 4 requires 66.47%, 79.26%, and 59.57% less area compared to [23],[3], and [11], respectively. It has 21.67%, and 31.74% less delay compared to [23], and [3], respectively. It consumes 78.69%, 66.71% and 58.94% less power than [23], [3], and [11], respectively. It has 73.72%, 85.83%, and 48.44% less ADP than [23], [3], and [11] respectively. It has 83.31%, 77.27%, and 47.78% less PDP compared to [23], [3], and [11] respectively.

## Conclusions

This paper implements two novel symmetry 2-D block FIRAs using QSF and DSF and one general filter (without symmetry) with DPMLUT-based multipliers. The conventional multipliers are replaced with the MLUT multipliers; DPMLUT-based multipliers save power and area compared to SPMLUT-based multipliers. Individual symmetry filters are implemented using fewer multipliers. Block processing is used to achieve memory reuse.

The proposed MLUT-based 2-D block DSF filter for L = 8 with N = 4 requires 54.79% less area, consumes 54.49% less power, has 41.72% less ADP, and 41.24% less PDP, but has 29% more delay compared to existing HLUT-based 2-D block FIR filter. ^11^

On the other hand, the proposed MLUT-based 2-D block QSF filter for L = 8 with N = 4 requires 59.5% less area, consumes 58.94% less power, 48.44% less ADP and 47.78% less PDP, but has 27% more delay compared to existing HLUT-based 2-D block FIR filter. ^11^ The 2-D block FIRA using QSF has fewer unique coefficients than the 2-D block FIRA using DSF. Hence, QSF performs well in terms of performance metrics.

## Data Availability

Figshare: Untitled ItemFIR FILTER. https://doi.org/10.6084/m9.figshare.22058801.v1. ^27^ This project contains the following underlying data:
- Data set.xlsx (testbench which defines the operational relation between input and output, results for different values of block length and also the results table in which existing and proposed models be compared). - larea_DSF.rep (area report of DSF_2D FIR filter, a tool generated area report downloaded while executing). - larea_QSF.rep (area report of QSF_2D FIR filter, a tool generated area report downloaded while executing). - lpower_DSF.rep (power report of DSF_2D FIR filter, a tool generated area report downloaded while executing). - lpower_QSF.rep (power report of QSF_2D FIR filter, a tool generated area report downloaded while executing). - ltiming_DSF.rep (timing report of DSF_2D FIR filter, a tool generated area report downloaded while executing). - ltiming_QSF.rep (timing report of QSF_2D FIR filter, a tool generated area report downloaded while executing). Data are available under the terms of the Creative Commons Attribution 4.0 International license (CC-BY 4.0).

## Software availability

Source code available from: https://github.com/pratyushachowdarich/2DFIR_FILTER.git

Archived source code at time of publication: https://doi.org/10.5281/zenodo.7631743. ^28^

license: GPL-3.0-only

## References

[1] Parhi KK : *VLSI Digital Signal Processing Systems: Design and Implementation, ch. 7.* New York: Wiley;1999.

[2] Sid-Ahmed MA : *Image Processing: Theory, Algorithms, and Architectures.* NewYork: McGraw-Hill;1995.

[3] Mohanty BK Meher PK Amira A : Memory Footprint Reduction for Power-Efficient Realization of 2-D Finite Impulse Response Filters. *IEEE Trans. Circuits Syst. I.* 2014;61(1):120–133. 10.1109/TCSI.2013.2265953

[4] Mohanty BK Al-Maadeed S Amira A : Systolic architecture for hardware implementation of two-dimensional non-separable filter-bank. *2013 8th IEEE Design and Test Symposium, IEEE.* 2013; pp.1–6.

[5] Venkata Krishna O Venkata Narasimhulu C Satya Prasad K : Implementation of Low Power and Memory Efficient 2D FIR Filter Architecture. *Int. J. Recent Technol. Eng.* 2019;8(1):927–935.

[6] Vinitha CS Sharma RK : New approach to low-area, low-latency memory-based systolic architecture for FIR filters. *J. Inf. Optim. Sci.* 2019;40(2):247–262. 10.1080/02522667.2019.1578087

[7] Chen PY Van LD Khoo IH : Power-efficient and cost-effective 2-D symmetry filter architectures. *IEEE Trans. Circuits Syst. I: Regular Papers.* 2010;58(1):112–125.

[8] Van LD Khoo IH Chen PY : Symmetry incorporated cost-effective architectures for two-dimensional digital filters. *IEEE Circuits Syst. Mag.* 2019;19(1):33–54. 10.1109/MCAS.2018.2872665

[9] Aggarwal A Kumar M Rawat TK : Design of two-dimensional FIR filters with quadrantally symmetric properties using the 2D L 1-method. *IET Signal Processing.* 2018;13(3):262–272.

[10] Mohanty BK Meher PK Singhal SK : A high-performance VLSI architecture for reconfigurable FIR using distributed arithmetic. *Integration.* 2016;54:37–46. 10.1016/j.vlsi.2016.01.006

[11] Kumar P Shrivastava PC Tiwari M : High-throughput, area-efficient architecture of 2-D block FIR filter using distributed arithmetic algorithm. *Circuits, systems, and signal processing.* 2019;38(3):1099–1113. 10.1007/s00034-018-0897-2

[12] Kumar P Shrivastava PC Tiwari M : ASIC implementation of area-efficient, high-throughput 2-D IIR filter using distributed arithmetic. *Circuits, Systems, and Signal Processing.* 2018;37(7):2934–2957. 10.1007/s00034-017-0698-z

[13] NagaJyothi G Sridevi S : High speed and low area decision feed-back equalizer with novel memoryless distributed arithmetic filter. *Multimed. Tools Appl.* 2019;78(23):32679–32693. 10.1007/s11042-018-7038-6

[14] NagaJyothi G Sridevi S : Distributed arithmetic architectures for fir filters-a comparative review. *2017 International conference on wireless communications, signal processing and networking (WiSPNET), IEEE.* 2017; pp.2684–2690.

[15] Park SY Meher PK : Efficient FPGA and ASIC realizations of a DA-based reconfigurable FIR digital filter. *IEEE Trans. Circuits Syst. II Exp. Briefs.* 2014;61(7):511–515.

[16] Venkata Krishna O Venkata Narasimhulu C Satya Prasad K : Efficient VLSI architectures for FIR Filters. *IOSR Journal of VLSI and Signal Processing.* 2016;6(6):37–44.

[17] Odugu VK Venkata Narasimhulu C Satya Prasad K : Implementation of Low Power Generic 2D FIR Filter Bank Architecture Using Memory-based Multipliers. *J. Mob. Multimed.* 2022;583–602. 10.13052/jmm1550-4646.1836

[18] Meher PK : New approach to look-up-table design and memory-based realization of FIR digital filter. *IEEE Trans. Circuits Syst. I: Regular Papers.* 2009;57(3):592–603. 10.1109/TCSI.2009.2026683

[19] Meher PK : New look-up-table optimizations for memory-based multiplication. *Proceedings of the 2009 12th International Symposium on Integrated Circuits. IEEE.* 2009; pp.663–666.

[20] Vinitha CS Sharma RK : An efficient LUT design on FPGA for memory-based multiplication. *Iran. J. Electr. Electron. Eng.* 2019;15(4):462–476.

[21] Chiper DF Swamy MS Ahmad MO : Systolic algorithms and a memory-based design approach for a unified architecture for the computation of DCT/DST/IDCT/IDST. *IEEE Trans. Circuits Syst. I: Regular Papers.* 2005;52(6):1125–1137. 10.1109/TCSI.2005.849109

[22] Sharma D Johnson J Sharma A : Memory-based FIR digital filter using modified OMS-LUT design. *Applications of Computing, Automation and Wireless Systems in Electrical Engineering.* Singapore: Springer;2019; pp.1007–1017. 10.1007/978-981-13-6772-4_88

[23] Alawad M Lin M : Memory-Efficient Probabilistic 2-D Finite Impulse Response (FIR) Filter. *IEEE Transactions on Multi-Scale Computing Systems.* 2017;4(1):69–82.

[24] Chowdari CP Seventline JB : An efficient FIR Filter Architecture Implementation using Distributed Arithmetic (DA) for DSP Applications. *International journal of Innovative Technology and Exploring Engineering.* 2019.

[25] Chowdari CP Seventline JB : Systolic architecture for adaptive block FIR filter for throughput using distributed arithmetic. *Int. J. Speech Technol.* 2020;23(3):549–557. 10.1007/s10772-020-09745-4

[26] Chowdari CP Seventline JB : VLSI implementation of distributed arithmetic-based block adaptive finite impulse response filter. *Materials Today: Proceedings.* 2020;33:3757–3762.

[27] ch, pratyushachowdari: Untitled ItemFIR FILTER.[Dataset]. *figshare.* 2023. 10.6084/m9.figshare.22058801.v1

[28] pratyusha chowdari ch: FIR filter [Software]. *Zenodo.* 2023. 10.5281/zenodo.7631743

