# Supplementary material for: Implementation of distributed arithmetic-based symmetrical 2-D block finite impulse response filter architectures
Source: F1000Res. 2023 Sep 21;12:1182. [Version 1] doi: 10.12688/f1000research.126067.1 (PMC10911408; doi:10.12688/f1000research.126067.1)
Supplement: Peer review history

# Reviewer report: Reviewer response for version 1

**DOI:** 10.5256/f1000research.138442.r242160

**Published:** 2024-02-29

**Authors:** M Balaji

**Affiliations:** 1 Department of ECE, Sree Vidyanikethan Engineering College, Tirupati, Andhra Pradesh, India

This study focuses on symmetry-based optimizations and offers a well-organized and very effective method for designing 2-D finite impulse response (FIR) filters. It is a good method to decrease multipliers by incorporating different symmetries. One notable innovation is the multiplication process that uses distributed arithmetic (DA) and the dual-port memory-based lookup table (DP-MLUT). It increases the FIR filter's overall efficiency while also consuming less space and power.

 One ingenious way to lower the requirement for registers and, hence, circuit complexity is to implement memory reuse and memory sharing solutions. The suggested architectures' practicality is greatly enhanced by this method. The benefits of the suggested designs in terms of power, area, area delay product (ADP), and power delay product (PDP) are well demonstrated by the comparison data presented in the study. These are significant advancements that have potential for many different uses.

 The synthesis produces a 45 nm technological environment that demonstrates how the suggested designs may be used in practical settings. Particularly with the Quadrantal Symmetry Filter (QSF), the power and area savings are very noteworthy. The appropriateness of the suggested filter design for applications with fixed coefficients is emphasized in the paper's conclusion. This understanding of possible uses gives the research more usefulness.

 Request the Corresponding author to provide justifications for these queries.

 1) Please explain how the area (micrometers square) was calculated.

 2) The proposed filter was designed with L=8 and N=4, it can be further extended to 32-bit with 64 taps.

 3) If the comparisons of your proposed results with existing results within a table format is appreciable."

Is the work clearly and accurately presented and does it cite the current literature?

Yes

If applicable, is the statistical analysis and its interpretation appropriate?

Yes

Are all the source data underlying the results available to ensure full reproducibility?

Yes

Is the study design appropriate and is the work technically sound?

Yes

Are the conclusions drawn adequately supported by the results?

Yes

Are sufficient details of methods and analysis provided to allow replication by others?

Yes

Reviewer Expertise:

FIR FIlter Design, Distributed Arithmetic, Residue Number System, VLSI Signal Processing

I confirm that I have read this submission and believe that I have an appropriate level of expertise to confirm that it is of an acceptable scientific standard.

## References

[1] : Area and delay efficient RNS-based FIR filter design using fast multipliers. *Measurement: Sensors* .2024;31: 10.1016/j.measen.2023.101014 10.1016/j.measen.2023.101014

[2] : Low power residue number system using lookup table decomposition and finite state machine based post computation. *Indonesian Journal of Electrical Engineering and Computer Science* .2022;26(1) : 10.11591/ijeecs.v26.i1.pp127-134 10.11591/ijeecs.v26.i1.pp127-134

[3] : High-Speed DSP Pipelining and Retiming techniques for Distributed-Arithmetic RNS-based FIR Filter Design. *WSEAS TRANSACTIONS ON SYSTEMS AND CONTROL* .2022;17: 10.37394/23203.2022.17.60 549-556 10.37394/23203.2022.17.60

[4] : A low power transistor level FIR filter implementation using CMOS 45 nm technology. *International Journal of Nanotechnology* .2023;20(1/2/3/4) : 10.1504/IJNT.2023.131120 390-409 10.1504/IJNT.2023.131120

# Reviewer report: Reviewer response for version 1

**DOI:** 10.5256/f1000research.138442.r208433

**Published:** 2023-11-01

**Authors:** Sirish Kumar Pagoti

**Affiliations:** 1 Department of ECE, Aditya Institute of Technology and Management, Srikakulam, Andhra Pradesh, India

1. This paper presents a well-structured and highly efficient approach to 2-D finite impulse response (FIR) filter design, focusing on symmetry-based optimizations. The incorporation of various symmetries to minimize multipliers is a commendable strategy.

 2. The utilization of distributed arithmetic (DA) and the dual-port memory-based lookup table (DP-MLUT) in multiplication is a noteworthy innovation. It not only reduces area and power consumption but also enhances the overall efficiency of the FIR filter.

 3. The introduction of memory reuse and memory sharing methods is a clever technique to reduce the need for registers and, consequently, circuit complexity. This approach contributes significantly to the practicality of the proposed architectures.

 4. The comparative results provided in the paper clearly demonstrate the advantages of the proposed architectures in terms of power, area, area delay product (ADP), and power delay product (PDP). These improvements are substantial and hold promise for a wide range of applications.

 5. The synthesis results in a 45nm technology environment showcase the real-world applicability of the proposed architectures. The power and area savings, especially in the Quadrantal Symmetry Filter (QSF), are quite impressive.

 6. The paper's conclusion emphasizes the suitability of the proposed filter architecture for applications with fixed coefficients. This insight into potential applications adds practical value to the research.

 7. Overall, this paper offers a valuable contribution to the field of FIR filter design. The innovative approaches, clear presentation, and substantial improvements in efficiency make it a significant advancement in the area of signal processing.

Is the work clearly and accurately presented and does it cite the current literature?

Yes

If applicable, is the statistical analysis and its interpretation appropriate?

Yes

Are all the source data underlying the results available to ensure full reproducibility?

Yes

Is the study design appropriate and is the work technically sound?

Yes

Are the conclusions drawn adequately supported by the results?

Yes

Are sufficient details of methods and analysis provided to allow replication by others?

Yes

Reviewer Expertise:

Communication, signal processing, wireless networks, embedded system

I confirm that I have read this submission and believe that I have an appropriate level of expertise to confirm that it is of an acceptable scientific standard.
